# Successful Delivery of Small Non-Coding RNA Molecules into Human iPSC-Derived Lung Spheroids in 3D Culture Environment

**DOI:** 10.3390/biomedicines13102419

**Published:** 2025-10-03

**Authors:** Anja Schweikert, Chiara De Santi, Xi Jing Teoh, Frederick Lee Xin Yang, Enya O’Sullivan, Catherine M. Greene, Killian Hurley, Irene K. Oglesby

**Affiliations:** 1 Department of Medicine, Education and Research Centre, Royal College of Surgeons in Ireland, D09YD60 Dublin, Ireland; anjaschweikert@rcsi.com (A.S.);; 2 Tissue Engineering Research Group, Royal College of Surgeons in Ireland, D02YN77 Dublin, Ireland; 3 School of Pharmacy and Biomolecular Sciences, Royal College of Surgeons in Ireland, D02YN77 Dublin, Ireland; chiaradesanti@rcsi.ie (C.D.S.);; 4 Department of Clinical Microbiology, Education and Research Centre, Royal College of Surgeons in Ireland, D09YD60 Dublin, Ireland

**Keywords:** lung spheroids, siRNA, miRNA, transient transfection, induced pluripotent stem cells

## Abstract

**Background/Objectives**: Spheroid cultures in Matrigel are routinely used to study cell behaviour in complex 3D settings, thereby generating preclinical models of disease. Ideally, researchers would like to modulate gene expression ‘in situ’ for testing novel gene therapies while conserving the spheroid architecture. Here, we aim to provide an efficient method to transfect small RNAs (such as microRNAs and small interfering RNAs, i.e., siRNAs) into human induced pluripotent stem cell (iPSC)-derived 3D lung spheroids, specifically alveolar type II epithelial cells (iAT2) and basal cell (iBC) spheroids. **Methods**: Transfection of iAT2 spheroids within 3D Matrigel ‘in situ’, whole spheroids released from Matrigel or spheroids dissociated to single cells was explored via flow cytometry using a fluorescently labelled siRNA. Validation of the transfection method was performed in iAT2 and iBC spheroids using siRNA and miRNA mimics and measurement of specific target expression post-transfection. **Results**: Maximal delivery of siRNA was achieved in serum-free conditions in whole spheroids released from the Matrigel, followed by whole spheroids ‘in situ’. ‘In situ’ transfection of *SFTPC*-siRNA led to a 50% reduction in the *SFTPC* mRNA levels in iAT2 spheroids. Transfection of miR-29c mimic and miR-21 pre-miR into iAT2 and iBC spheroids, respectively, led to significant miRNA overexpression, together with a significant decrease in protein levels of the miR-29 target FOXO3a. **Conclusions**: This study demonstrates successful transfection of iPSC-derived lung spheroids without disruption of their 3D structure using a simple and feasible approach. Further development of these methods will facilitate functional studies in iPSC-derived spheroids utilizing small RNAs.

## 1. Introduction

Recent advances in stem cell technology have produced novel in vitro model systems derived from patient somatic cells, which can be ‘reprogrammed’ into induced pluripotent stem cells (iPSCs) [1]. iPSCs can then be directed to differentiate towards a specific cell fate and grown as self-organizing cell clusters, termed ‘spheroids’, in 3D cell culture systems. iPSC-derived spheroids provide a unique model for studying human pathobiology, as they can grow into 3D structures that closely resemble the function of the native organ they are fated towards. Importantly, iPSC-derived spheroids can model hard-to-access tissue environments, such as the proximal and distal lung, providing an ideal platform for the study of several lung diseases, including cystic fibrosis (CF) [2,3] and idiopathic pulmonary fibrosis (IPF) [4,5].

While the extracellular matrix (ECM) embedding the spheroids is crucial to mimic the tissue environment, it may also hinder any transfer of genetic material into them. Moreover, certain spheroid models consist of compact structures, which further obstruct the diffusion, penetration, and cellular uptake of genetic material. Nonetheless, transfection of spheroids has been achieved using electroporation [6,7,8], liposome-based transfection reagents [9,10,11] and, more recently, a ‘transfection reagent-free’ method [12]. While these pilot reports have shown significant delivery of small interfering RNA (siRNA) or plasmids of interest, they also present with some limitations, including uneven uptake of siRNA within the 3D structure and the need to remove the spheroids from the Matrigel. This often involves the complete dissociation of spheroids into single cells prior to genetic manipulation. In the case of transient gene expression studies, the impact of miRNA or siRNA manipulation in spheroids dissociated into single cells may differ significantly from that of spheroids in their 3D format, or be lost by the time the formation of 3D spheroids post-transfection occurs.

Importantly, none of these studies were conducted on iPSC-derived spheroids; therefore, efficient transfection methods for these models are currently lacking. Herein, we used two iPSC-derived spheroid models of distal and proximal lung, respectively, i.e., alveolar type II epithelial cell (iAT2) spheroids [13,14] and induced airway basal cell (iBC) spheroids [15]. We first examined transient siRNA transfection of iAT2 spheroids under three conditions: (i) whole spheroids ‘in situ’ within 3D Matrigel; (ii) whole spheroids dislodged from Matrigel and transfected prior to re-plating in 3D Matrigel; and (iii) spheroids dissociated to single cells, transfected, and then re-plated in 3D Matrigel. We compared these three methods in terms of transfection efficiency (via flow cytometry) and cytotoxicity (via lactate dehydrogenase (LDH) assay) by using a commercially available transfection reagent and a fluorescently labelled siRNA. SiRNAs regulate gene expression via the RNA-induced silencing complex (RISC), which leads to gene silencing, and are widely used as a tool to knock down target genes [16]. Therefore, we tested that the efficient uptake of the siRNA led to the knock down of its target mRNA, by transfecting surfactant protein C (*SFTPC*) siRNA into iAT2 spheroids ‘in situ’ within 3D Matrigel and measuring its mRNA expression after transfection. Additionally, we extended this method to examine transfection of another type of small non-coding RNA molecule, namely microRNA (miRNA). MiRNAs act as post-transcriptional regulators in virtually all known biological processes in human cells, where they induce mRNA degradation or translational repression of their target genes [17]. Here, we transfected an miR mimic of miR-29c, known to be downregulated in IPF [18], into iAT2 spheroids and measured the expression of its target forkhead box O3a (FOXO3a3) [18] as an indication of transfection efficiency. Finally, we expanded upon the data we previously generated on miRNA transfection in iPSC-derived CF Airway Epithelial spheroids [19] by applying the ‘in situ’ transfection approach for miRNA transfection in further matured CF iBC spheroids [15].

## 2. Materials and Methods

### 2.1. Spheroid Cell Culture

All cells were cultured in a humidified incubator at 37 °C with 5% CO_2_.

### 2.2. Distal Lung Spheroids (iAT2 Spheroids)

Non-diseased iAT2 spheroids were generated using a previously published directed differentiation protocol [13,14] from the male, disease-corrected SPC2-ST-B2 iPSC line kindly donated by Prof. Darrell Kotton (Center for Regenerative Medicine, Boston University). This iPSC line contains a tdTomato reporter in the promoter region of the canonical AT2 cell marker, SFTPC, which can be used to isolate a pure population of AT2 cells. iAT2 spheroids were maintained in 3D Matrigel in ‘CK+DCI’ media, consisting of complete serum-free dissociation media (cSFDM) base supplemented with 3 mM CHIR99021 (Tocris, Bristol, UK), 10 ng/mL rhKGF (R&D Systems, Minneapolis, MN, USA) (CK), 50 nM dexamethasone (Sigma, Darmstadt, Germany), 0.1 mM 8-Bromoadenosine 30,50-cyclic monophosphate sodium salt (Sigma), and 0.1 mM 3-Isobutyl-1-methylxanthine (IBMX) (Sigma) (DCI). iAT2 spheroids were passed every 10–14 days at a concentration of 500 cells/μL. The transfection experiments were carried out at 80–90% confluency.

### 2.3. Proximal Lung Spheroids (iBC Spheroids)

iBC spheroids were generated using a modified version of the previously published proximal lung-directed differentiation protocol [15]. Briefly, iPSCs from an individual with CF homozygous for the Phe508del mutation (F508del/F508del) were differentiated towards early lung progenitors using STEMdiff^TM^ Lung Progenitor Kit (StemCell Technologies, Vancouver, BC, Canada). On day 14 of differentiation, live cells were sorted based on magnetic-activated cell sorting (MACS) using the Miltenyi Biotec MiniMACS Separator in combination with anti-mouse IgG(H+L) MicroBeads to isolate the NKX2-1+ lung progenitors based on carboxypeptidase M (CPM) as a surface marker of NKX2-1 [20,21]. The sorted lung progenitors were seeded in growth factor-reduced 3D Matrigel (Corning, Corning, NY, USA) at a concentration of 500 cells/µL. Proximal lung differentiation of cells was performed in ‘’Airway Differentiation Medium’’ media consisting of cSFDM base supplemented with 250 ng/mL rhFGF2 (R&D Systems), 100 ng/mL rhFGF10 (R&D Systems), 50 nM dexamethasone, 0.1 mM 8-Bromoadenosine 30,50-cyclic monophosphate sodium salt, and 0.1 mM IBMX (DCI). On day 30 of the directed differentiation, culture medium was switched to Pneumacult ExPlus Basal Medium (Stemcell Technologies), supplemented with 1µM A83-01 (Tocris), 1 µM DMH1 (Tocris), and 10 µM Y-27632 (Tocris). Airway Epithelial spheroid cells were MACS-sorted based on the basal cell marker nerve growth factor receptor (NGFR) on Day 42 and seeded as described above. The resulting iBC (day 60+) spheroids were used for transfection experiments.

### 2.4. Transfection of iPSC-Derived Lung Spheroids with Fluorescently Labelled siRNA

**Figure 1:**
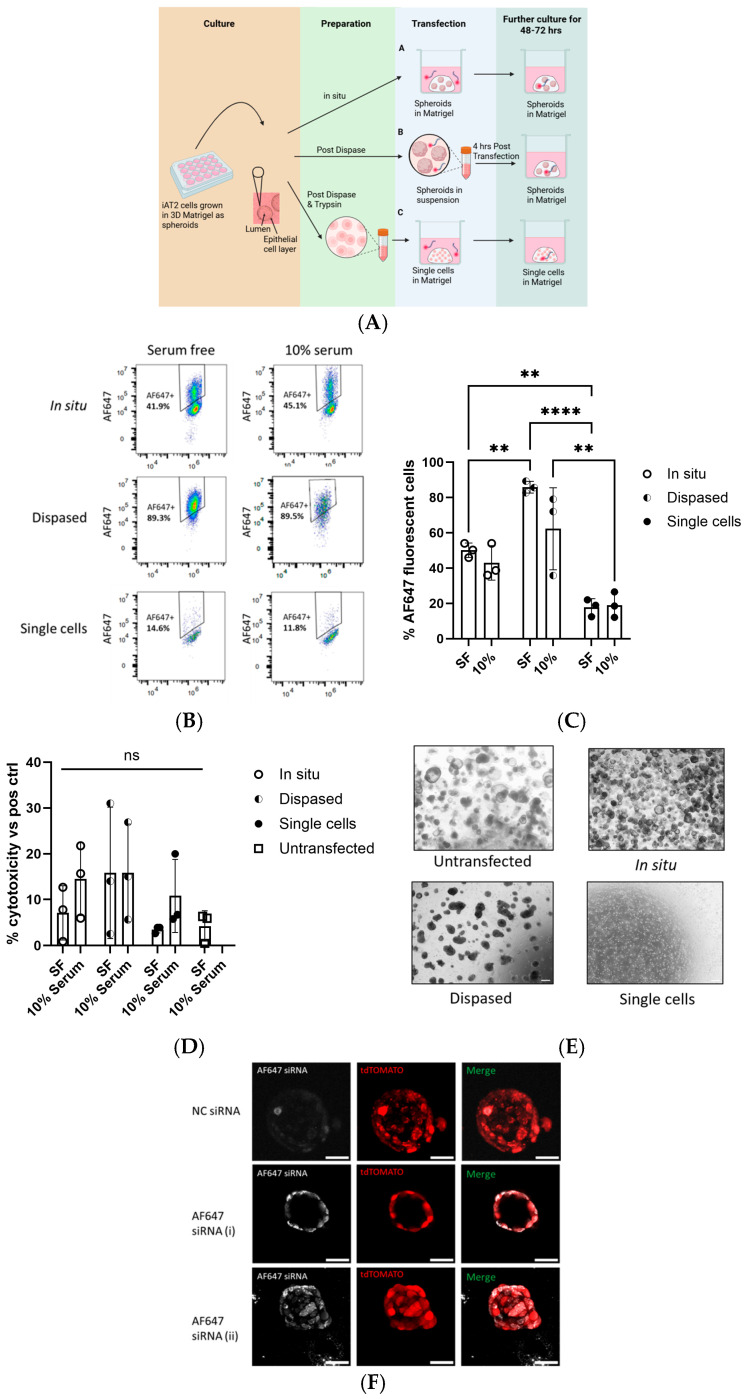
iAT2 spheroids are efficiently transfected ‘in situ’ using Ribojuice. (**A**) Outline of the different transfection protocols: Briefly, iAT2 spheroids were transfected either as spheroids within 3D Matrigel ‘in situ’ (top panel), post-dispase spheroids in suspension (middle panel), or post-dispase and trypsin and re-seeded into Matrigel as single cells (bottom panel). (**B**) Representative flow cytometry graph of the three transfection methods, in the absence or presence of 10% FBS. (**C**) Quantification of the flow cytometry data: % of AF647-positive cells for the samples transfected with fluorescently labelled siRNA are reported (*n* = 3 independent experiments, in triplicate). Data are expressed as mean ± SD, and *p*-values are reported according to Sidak’s multiple comparisons test (two-way ANOVA). (**D**) Cytotoxicity of the three transfection methods as measured via lactate dehydrogenase (LDH) assay (*n* = 3 independent experiments, in triplicate). Data are expressed as mean ± SD, and *p*-values are reported according to Sidak’s multiple comparisons test (two-way ANOVA). (**E**) Representative bright-field image of non-transfected and transfected spheroids using the three different approaches. (**F**) Image of whole iAT2 spheroids transfected with fluorescently labelled negative control (NC) siRNA or AF647 siRNA and imaged by confocal microscopy. White indicates the expression of AF647 siRNA and red represents tdTOMATO (i.e., AT2 cells). Scale bar: 50 µm. Two images, (i) and (ii), for the AF647 siRNAs are shown to represent the different shapes the spheroids can take in 3D cultures. *p*-values are reported on the graph as following: ns = not significant, ** *p* < 0.01, **** *p* < 0.0001.

iAT2 spheroids were used to assess the transfection efficiency and cytotoxicity of different transfection methods. Three transfection methods, all using the commercially available transfection reagent Ribojuice (3.5 µL/well of a 24-well plate) (Merck) and a fluorescently labelled non-targeting siRNA (AllStars Neg. siRNA AF647, Qiagen, Hilden, Germany) or its non-fluorescently labelled counterpart (DNeg ctrl siRNA, IDT, Coralville, IA, USA) (50 nM), consisted of the following: (i) ‘in situ’ transfection of whole spheroids, where the spheroids remained undisturbed in the 3D Matrigel, with the transfection mix being added dropwise into the culture media; (ii) transfection of ‘dispased’ spheroids, where the spheroids were gently dislodged from the 3D Matrigel using Dispase II, transferred to a 1.5 mL microfuge tube for 4 h for the transfection process, and then re-plated into 3D Matrigel; (iii) ‘single cell’ transfection, where the spheroids were dislodged from the 3D Matrigel using Dispase II, dissociated into single cells using 0.05% Trypsin, re-plated into 3D Matrigel, and the transfection mix was added directly into the culture media dropwise. Transfection mixes were prepared in CK+DCI media, in the presence or absence of 10% FBS, and supplemented with 10 µM Y-27632. A schematic representation of the three methods used is shown in Figure 1A. Cells were maintained in the transfection medium for 48–72 h, depending on downstream analysis.

Transfection of iBC spheroids, performed as above (i.e., ‘in situ’ transfection of the AF647-labelled siRNA), was also examined.

### 2.5. Flow Cytometry

Following transfection for 48 h, cells were removed from Matrigel using Dispase II and dissociated to single cells using Trypsin for assessment of transfection efficiency by flow cytometry. Briefly, single cells were filtered and resuspended in FACS Buffer (1% FBS in PBS without Ca^2+^ and Mg^2+^ supplemented with 10 µM Y-27632) containing 10 µM calcein blue (Thermofisher Scientific, Waltham, MA, USA) to label live cells. AF647-positive cells (i.e., transfected cells) were analysed by flow cytometry using CytoFlex S (Becton Dickenson, Franklin Lakes, NJ, USA). Fluorescence signal was detected using violet laser 1 (filter wavelength: 440/50; PB450 for calcein blue signal) and red laser 1 (filter wavelength: 670/14; APC for AF647 signal). Representative flow cytometry gating strategy is shown in Supplemental Figure S1. Data were analysed using flow cytometry software FlowJo V10 (BD Biosciences). Percentage of median fluorescence intensity (MFI) of AF647+ cells (i.e., transfected with fluorescently labelled siRNA) was determined for each sample.

### 2.6. Cytotoxicity/LDH Assay

LDH release was measured using the CytoTox 96 Non-Radioactive Cytotoxicity Assay (Promega, Madison, WI, USA) according to the manufacturer’s instructions at 48 h post-transfection. Briefly, positive controls were generated from the cells in an untransfected well by adding the 10× lysis solution provided in the assay kit directly into the well for 45 min at 37 °C. Fifty µL of supernatant was collected from transfected cells from each of the three methods used and the positive control, and incubated with 50 µL Substrate Solution for 30 min at RT. After incubation, 50 μL of Stop Solution was added per well. The absorbance was measured at 490 nm on a Wallac Victor2 plate reader (Perkin Elmer, Waltham, MA, USA). The percentage of cytotoxicity was determined by dividing the experimental LDH release by the maximum LDH release from the positive controls, after background (medium only) subtraction, and multiplying the resulting value times 100.

### 2.7. Confocal Microscopy

iAT2 spheroids were seeded at a density of 500 cells/μL on glass cover slips in 6-well plates and maintained in CK+DCI for 10–11 days until 80–90% confluency was reached. They were then ‘in situ’ transfected with the negative control (NC) siRNA or AF647 siRNA (50 nM) using Ribojuice transfection reagent for 72 h. At 72 h post-transfection, single images of the transfected spheroids were acquired on a Carl Zeiss LSM 710 confocal microscope (Carl Zeiss, Jena, Germany) using W Plan-Apochromat 20×/1.0 water immersion objective. The tdTomato was excited using a 561 nm laser (detection range: 564–629 nm). The siRNA fluorescence was excited using a 633 nm laser (detection range: 637–685 nm). All the images were prepared in FIJI [22].

### 2.8. siRNA and miRNA Studies

iAT2 spheroids were ‘in situ’ transfected with NC or *SFTPC* siRNA (hs.Ri.SFTPC.13.1), and with miRVana™ miRNA Mimic miR-29c-3p (Assay ID MC10518, Thermofisher Scientific, Waltham, MA, USA) or Negative Control #1 (Thermofisher Scientific). iBC spheroids were ‘in situ’ transfected with miR-21 pre-miR (Assay ID PM10206) or pre-miR Negative Control #1 (Thermofisher Scientific). All mimics or siRNAs were transfected at a final concentration of 50 nM for 48 h.

### 2.9. Gene Expression Analysis—Quantitative Real-Time Polymerase Chain Reaction (qRT-PCR)

At 48 h post-transfection, transfected cells were dissociated from the 3D Matrigel using Dispase II. Cell pellets were resuspended in 500 µL of Tri-Reagent (Sigma) and RNAs were extracted according to the manufacturer’s protocol.

For the miRNA analysis, 20 ng of total RNA was reverse transcribed using the Applied Biosystems TaqMan™ microRNA reverse transcription kit (Thermofisher Scientific, Cat. 4366596) and the following miRNA-specific TaqMan MicroRNA Assays (Cat. 4427975 Thermofisher Scientific): miR-29c-3p (Assay ID 000587), miR-21 (Assay ID 000397), and U6 snRNA (Assay ID 001973). The miRNA analysis was performed using TaqMan™ Fast Advanced Master Mix (Thermofisher Scientific, Cat. 4444557) according to manufacturer’s instructions using the LC480 LightCycler (Roche, Basel, Switzerland). Expression of miRNAs relative to U6 snRNA, used as the reference gene, was determined using the 2^(−ΔΔCt)^ method [23].

For the target mRNA analysis, equal quantities of RNA were reverse transcribed to cDNA using the QuantiTect Reverse Transcription kit (Qiagen). Expression of *SFTPC* (SFPTC_F: CACAACTTCCAGGCCAAGCC; SFTPC_R: GTCCTCACACTCTTGGCATAGG), *FOXO3a* (FOXO3A_F: CTACGAGTGGATGGTGCGTT; FOXO3A_R: TGTGCCGGATGGAGTTCTTC) and *PTEN* (PTEN_F: CTCCCAGACATGACAGCCATC; PTEN_R: TCAAAAGGATATTGTGCAACTCTGC) mRNAs relative to *Rplp0* (Rplp0_F: CCTCATATCCGGGGGAATGTG; Rplp0_R: GCAGCAGCTGGCACCTTATTG) or 18S (18S_F: GGATGTAAAGGATGGAAAATACA; 18S_R: TCCAGGTCTTCACGGAGCTTGTT), used as the reference genes, was determined using the 2^(−ΔΔCt)^ method [23] using SYBR green chemistry and the LC480 LightCycler (Roche).

### 2.10. Protein Analysis—Western Blotting

At 48 h post-transfection, cells were dissociated from the 3D Matrigel using Dispase II. Cell pellets were resuspended in 100 µL of low-stringency lysis buffer (50 mM HEPES [pH 7.5], 100 mM NaCl, 10% glycerol [*v*/*v*], 0.5% Nonidet P-40 [*v*/*v*], supplemented with EDTA-free Protease Inhibitor Cocktail (Roche)). The resulting suspension was centrifuged at 16,000× *g* for 20 min at 4 °C, and the supernatants were collected and used for SDS-PAGE. Protein samples were quantified by BCA protein assay (Pierce, Rockford, IL, USA), denatured by the addition of 4× SDS sample buffer containing 0.2 M DTT, and heated for 10 min at 95 °C. Equal amounts of whole-cell lysates (10–20 µg) were separated on 4–12% Bis-Tris acrylamide gels (Thermofisher Scientific), transferred to polyvinylidene difluoride membranes (Roche), and probed with mouse anti-FOXO3a (1:1000, cat no. ab109629, Abcam) or mouse anti-β-actin antibodies (1:1000, cat no. 3700S, Cell Signaling). Anti-mouse IgG, HRP-linked antibodies (1:2500, cat no. 7076S Cell Signaling) were used as a secondary antibody for one hour at RT. Detection was achieved using Immobilon Western Chemiluminescent HRP Substrate (Merck Millipore, Darmstadt, Germany), and the membranes were analysed by densitometry using ImageJ software (Version 1.54p). For the quantitative analysis, the signal intensity of each band was normalized to the β-actin densitometry values.

### 2.11. Statistical Analyses

Analyses were performed using GraphPad PRISM 10. Results were expressed as mean ± standard deviation (SD) and compared using two-tailed *t*-test or ANOVA, as appropriate and indicated in figure legends. Statistical significance was defined as ns = not significant, * = *p* < 0.05, ** = *p* < 0.01, *** = *p* < 0.001, and **** = *p* < 0.0001. Further details of statistical analyses and details of ‘n’ numbers, where ‘n’ represents number of independent experiments performed, are outlined in corresponding figure legends.

## 3. Results

### 3.1. iPSC-Derived AT2 Spheroids Can Successfully Be Transfected as Whole Spheroids in 3D Matrigel Without Serum Supplementation

In the transfection experiments using iAT2 spheroids, transfection efficiency of three different approaches (Figure 1A) was estimated as the %AF647-positive live cells, which represents the percentage of cells successfully transfected with AF647 fluorescent siRNA measured by flow cytometry (Figure 1B). There was no statistical difference in the %AF647-positive cells between serum-free (SF) and 10% FBS in ‘in situ’, ‘dispased’ spheroids, or single-cell transfections. The highest transfection efficiency, at 85.9 ± 3.3%, was observed in the SF-transfected, ‘dispased’ spheroids, followed by the ‘in situ’ spheroids at 50.1 ± 4.1%, and the single cells at 17.8 ± 4.9%, with statistical significance reached in all these comparisons (Figure 1C). The same trend was observed in the presence of 10% FBS, where again the ‘dispased’ spheroids displayed the highest transfection efficiency (62.3 ± 23.2%), followed by the ‘in situ’ spheroids (42.9 ± 9.7%) and single cells (19.0 ± 7.2%); however, only the difference between the ‘dispased’ and single cells reached statistical significance (Figure 1C).

The LDH assay results indicated that there was an increasing level of cytotoxicity in ‘dispased’ spheroids (both in the presence and absence of serum) and in ‘in situ’ or single-cell transfected spheroids (only in the presence of serum) when compared to the non-transfected control. However, none of these differences were statistically significant (Figure 1D).

Finally, we observed the morphology of the cells upon transfection (Figure 1E). The whole spheroids ‘in situ’ were not disrupted as transfection was achieved via the addition of transfection complexes directly into the culture media only, and the morphology remained unchanged in comparison to the non-transfected controls. The ‘dispased’ spheroids were dislodged from the 3D Matrigel prior to transfection in tubes for 4 h, followed by re-plating in 3D. Despite maintenance of a 3D structure, their morphology was significantly altered compared to the non-transfected spheroids, and they displayed a more irregular, misshapen form, losing their spherical appearance. Finally, the ‘single cells’ morphology remained the same pre- and post-transfection, without formation of whole spheroids within 48 h.

Overall, the flow cytometry data indicated that transfecting ‘dispased’ spheroids in the absence of serum was the most effective method to deliver small RNAs into iAT2 spheroids. Notably, the differences in the LDH levels amongst the methods were not significant, and this may have been due, at least in part, to the high variability we observed in the LDH readings between the experiments. As alternative to LDH assay as a measure of cytotoxicity, other parameters could be measured to compare the biocompatibility of the transfection methods, such as cell viability using tetrazolium-based assays (for example, MTS), apoptosis via caspase activity assays, or Annexin V flow. Nonetheless, we did observe some cytotoxicity (albeit not statistically significant) and morphological irregularities in the dispased compared to the ‘in situ’ transfected spheroids (Figure 1D,E). These findings, coupled with the highly laborious and more costly procedure of dislodging the spheroids from the Matrigel prior to transfection and subsequent re-plating after 4 h, led us to opt for the ‘in situ’ transfection in the absence of serum for further analyses, given that it achieved reasonable levels of transfection efficiency (≈50%), did not lead to significantly increased cytotoxicity, and did not disturb the 3D culture environment. Confocal microscopy confirmed that the siRNA molecules were effectively taken up by the spheroids when transfected ‘in situ’, and appeared to be concentrated towards the spheroids’ surfaces (Figure 1F).

### 3.2. Successful ‘In Situ’ SF Transfection of iAT2 and iBC Spheroids with siRNAs and miRNA Mimics

Following achievement of fluorescent siRNA uptake into iAT2 spheroids ‘in situ’, as demonstrated by flow cytometry and confocal microscopy, we investigated whether the 50% transfection efficiency we observed in the ‘in situ’ transfection condition (in the absence of serum) would translate into a corresponding downregulation of target gene expression upon transfection of siRNA or miRNA mimics.

**Figure 2:**
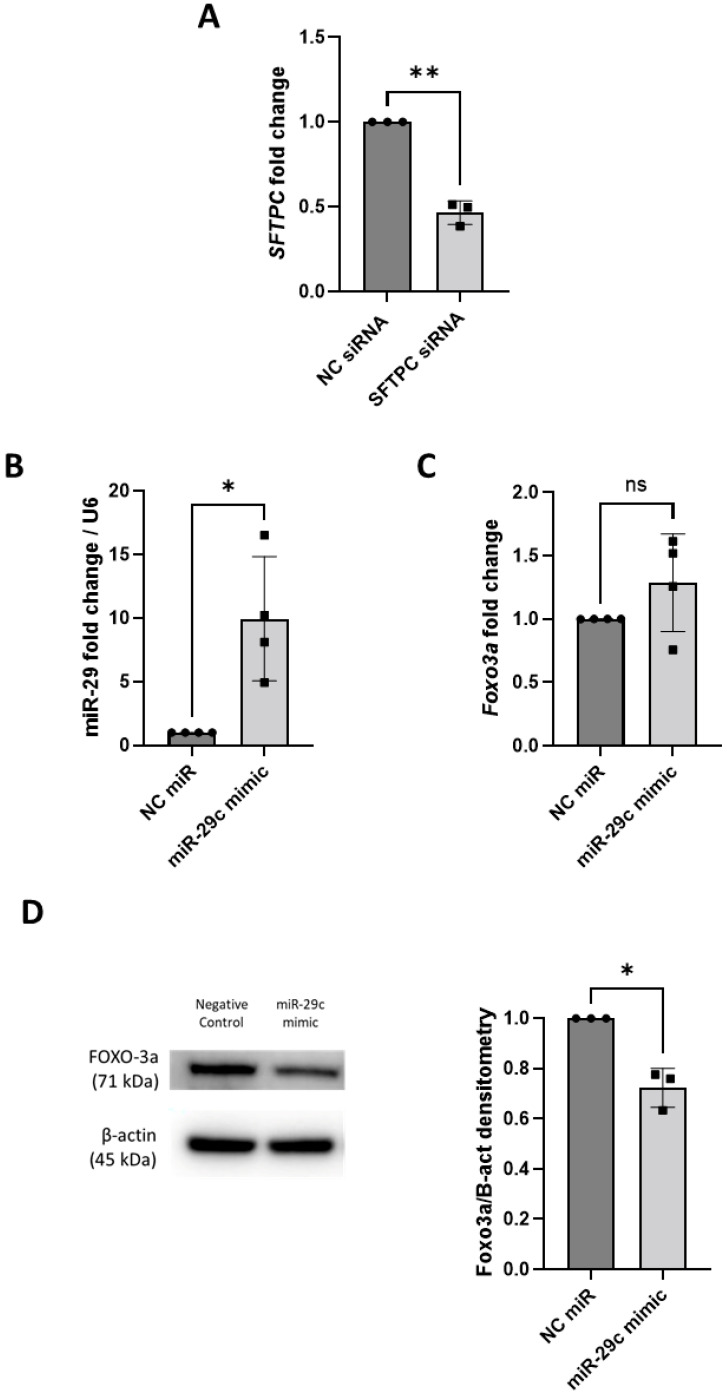
siRNAs and miRNAs can be transfected ‘in situ’ in iAT2 spheroids and decrease the expression of the target genes. (**A**) *SFTPC* mRNA levels measured after transfection of *SFTPC* siRNA (50 nM; 48 h) into iAT2 spheroids (*n* = 3 independent experiments, in triplicate). (**B**,**C**) miR-29c and its target *FOXO3a* levels and (**D**) FOXO3a protein expression measured after transfection of miR-29c mimics (50 nM; 48 h) into iAT2 spheroids (*n* = 3 independent experiments, at least, in triplicate for RNA; *n* = 3, in single for protein). Data are expressed as mean ± SD (NC siRNA-/mimic-transfected samples are set at 1). *p*-values are reported according to paired *t*-tests. *p*-values are reported on the graph as following: ns = not significant, * *p* ≤ 0.05, ** *p* < 0.01.

SFTPC is a surfactant protein essential for lung function and homeostasis after birth, and it is highly and exclusively expressed in AT2 cells [24]. Therefore, we used an *SFTPC* siRNA to evaluate the knock-down efficiency using the ‘in situ’ transfection protocol in the iAT2 spheroids. *SFTPC* mRNA levels were significantly decreased upon *SFTPC* siRNA transfection when compared to the NC siRNA (−53%, *p* = 0.0055) (Figure 2A).

MiR-29c has been implicated in apoptosis of AT2 cells by targeting *FOXO3a* mRNA [18]. Transfection of a miR-29c mimic ‘in situ’ into iAT2 spheroids resulted in a significant 8.9-fold increase in miR-29c expression (*p* = 0.035) compared to the NC mimic-transfected spheroids (Figure 2B). Overexpression of miR-29c did not lead to any change in *FOXO3a* at the mRNA level (Figure 2C); however, a significant decrease in FOXO3a protein was observed in the miR-29c-transfected iAT2 spheroids compared to the NC mimic-transfected ones (−27%, *p* = 0.025) (Figure 2D).

**Figure 3:**
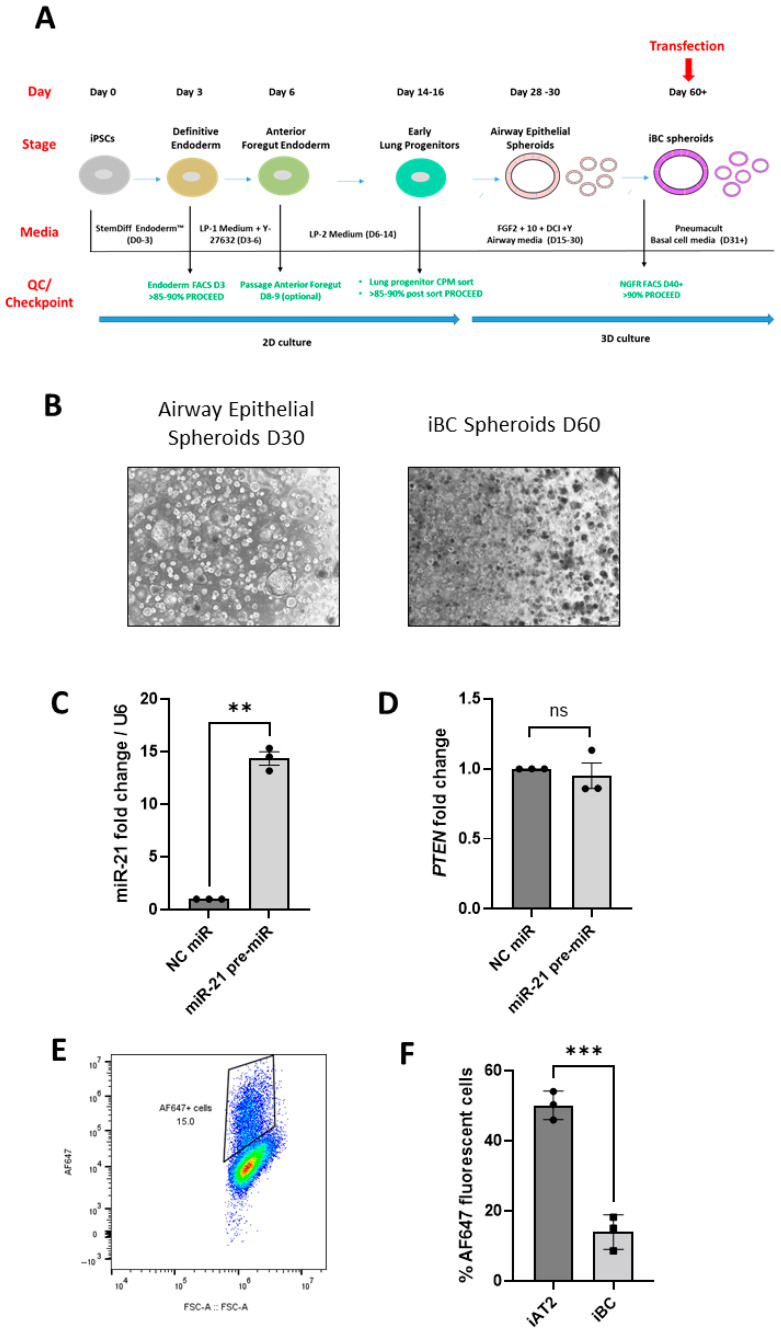
miRNAs can be transfected ‘in situ’ in iBC spheroids. (**A**) Schematic diagram of the iPSC differentiation towards iBC spheroids. The days of the protocol, stages of differentiation, media used, and quality control (QC)/checkpoints are highlighted in the figure. (**B**) Bright-field images of Airway Epithelial spheroids (Day 30 of the differentiation protocol; single-cell-layer spheroids) and iBC spheroids (Day 60 of the differentiation protocol; denser and more compact spheroids). (**C**) miR-21 and (**D**) *PTEN* mRNA levels measured after transfection of miR-21 pre-miR mimics (50 nM; 48 h) into iBC spheroids (*n* = 3 independent experiments, at least in duplicate). Data are expressed as mean ± SD (NC mimic-transfected samples are set at 1). *p*-values are reported according to paired *t*-tests. (**E**) Representative flow cytometry graph of the ‘in situ’ transfection efficiency in iBC spheroids. (**F**) Comparison of the % of AF647-positive cells upon transfection with fluorescently labelled siRNA of iAT2 (*n* = 3, in triplicate) vs. iBC spheroids (*n* = 3, in single). Data are expressed as mean ± SD. *p*-values are reported according to unpaired *t*-tests. *p*-values are reported on the graph as following: ns = not significant, ** *p* < 0.01, *** *p* < 0.001.

Finally, we sought to validate the ‘in situ’ transfection method in another iPSC-derived lung spheroid model, i.e., iBC spheroids (Figure 3A,B). We have reported a decrease in miR-21 expression in CF bronchial epithelium [25] and CF iBC spheroids [26], coupled with a significant increase in its known target *PTEN* [27]. Here, we observed a significant 13.3-fold increase in miR-21 levels (*p* = 0.002) in CF iBC spheroids when transfected ‘in situ’ with miR-21 pre-miR (Figure 3C). The expression of *PTEN* was not significantly altered upon transfection (Figure 3D); however, the ‘in situ’ transfection efficiency in this spheroid model was significantly reduced in comparison to that of the iAT2 spheroids (13.9% compared to 50.1% measured in iAT2 spheroids, *p* = 0.0007) with Ribojuice (Figure 3E,F).

Overall, these results indicate that the serum-free ‘in situ’ transfection method can successfully deliver small RNAs into iPSC-derived lung spheroids and has a knock-down effect on their downstream targets.

## 4. Discussion

The integration of stable genetic modification techniques, such as CRISPR-Cas9, into lung spheroid cultures has revolutionized disease modelling for studying lung development [28], as well as for investigating disease-causing genes relevant to regenerative medicine [29]. RNA interference (RNAi) is a commonly employed approach for loss-of-function studies and involves a siRNA or miRNA binding to its target mRNA via RISC and causing degradation of the mRNA or translational repression [30]. Methods for transient transfection of siRNAs and miRNAs into iPSC-derived spheroids without disturbing the 3D environment remain limited and under-reported. Here, we report for the first time a head-to-head comparison of different transfection approaches in human iPSC-derived spheroid models of distal (iAT2 spheroids) and proximal (iBC spheroids) lung, which range from a minimal (i.e., ‘in situ’ transfection) to complete (i.e., single-cells transfection) disruption of the 3D environment and the spheroids themselves. Whilst we found that the greatest level of transfection occurred in whole organoids removed from the 3D matrix, the spheroid architecture was impacted post-re-plating. In addition, this full disruption method was a more labour-intensive and costly (due to the need to re-plate the spheroids in 3D Matrigel post-transfection) procedure, leading to the adoption of the simpler ‘in situ’ method, for which we demonstrate a modest, but functionally relevant, transfection efficiency.

In this study, we found that iAT2 spheroids can be successfully transfected using an ‘in situ’ method, where the spheroids are maintained in 3D Matrigel and the transfection mix is added directly into the surrounding culture media. Employing this simple and effective approach, we observed an approximately 50% transfection efficiency as demonstrated by uptake of AF647 fluorescently labelled siRNA by flow cytometry. Using confocal microscopy, we found that the majority of fluorescence appeared to be concentrated in the spheroids towards the top of the Matrigel dome, suggesting that the uptake of siRNA was not uniform across the Matrigel drop; however, more in-depth analyses are required to assess the spatial distribution of the siRNA upon transfection. A similar approach to quantifying siRNA uptake via flow cytometry was employed by Pelofy et al., who transfected Cy5-labelled siRNAs into multicellular tumour spheroids via electroporation, and reported that 43% of the cells were positive for Cy5 [8]. The authors observed that siRNA uptake was not uniform across the spheroid, where better uptake and subsequent gene silencing was exhibited at the periphery rather than the core of the spheroids.

We found that the addition of serum into the transfection mix did not confer any advantage in terms of transfection efficiency or toxicity. This is in contrast to a recent study by Morgan et al., where serum addition improved siRNA penetration through 3D Matrigel and uptake into tumour spheroids and primary murine organoids [10]. However, the cells used in our study were routinely cultured under serum-free conditions and were exposed to serum in culture for the first time during the transfection process. This may explain their enhanced performance without serum and also suggests, overall, that differences in the transfection outputs in 3D cultures likely depend upon the cell type and routine culture methods.

Following ‘in situ’ transfection of a siRNA or miRNA mimic in the iAT2 spheroids, we observed a 50% reduction in the *SFTPC* mRNA and a 27% reduction in the FOXO3a protein upon *SFPTC* siRNA and miR-29c mimic transfection, respectively. In a recent study by Riching et al., a transfection reagent-free delivery of siRNA into tumour spheroids embedded in Matrigel or GrowDex resulted in 50–70% target knock-down [12]. However, in their protocol three doses of siRNA were given, each of which, at 2 µM, was 40 times greater than our single dose, with knock-down tested at 96–144 h post-transfection. Morgan et al. reported siRNA inhibition of *CTNNB1* mRNA (81%) using Lipofectamine 2000 or RNAiMax in tumour spheroids in the presence of 10% serum; however, this was not accompanied by a report of its effect on protein levels. We measured a significant reduction in the FOXO3a protein upon transfection of miR-29c mimic using serum-free conditions. These differences could be attributed to the composition of the different transfection reagents, whereby Morgan et al. employed liposome-based transfection reagents and we used Ribojuice, a non-liposomal cationic polymer/lipid mixture.

In an effort to validate our transfection approach in another iPSC-derived lung model, we overexpressed miR-21, which is known to be downregulated in CF [25], in CF iBC spheroids using miR-21 pre-miR. These iBCs were obtained using a previously published protocol [15], with a novel modification at the iBC sorting step, which was performed using MACS microbeads instead of fluorescence-activated cell sorting (FACS) to isolate the NGFR-positive cells. To the best of our knowledge, this is the first report of iBC generation utilizing MACS to enrich for basal cell marker nerve growth factor receptor (NGFR)-positive Airway Epithelial spheroid cells. Overexpression of miR-21 in the iBC cell spheroids did not result in reproducible inhibition of the known target *PTEN* mRNA, which may have been in part due to the reduced transfection efficiency observed in these cells. Due to the small numbers of iBC spheroids available for transfection at the end of the lengthy differentiation process, the PTEN protein levels were not measured on this occasion.

Taken together, our findings and prior studies indicate that the transfection efficiency in spheroid cultures can be impacted by the serum, dose of the small RNAs, experimental duration, choice of transfection reagents, and, importantly, the cell type/spheroid model. In addition, other methods, such as electroporation, could be used as an alternative to transfection reagents; however, this has mainly been used to transfect plasmids [6,9,31] rather than small RNAs.

With the growing importance of human ex vivo models for disease modelling, the simple transfection approach we present here will enable the investigation of miRNAs and siRNAs in iPSC-derived patient-specific spheroids as potential therapeutic strategies. SiRNAs are already being used as therapies, with the first siRNA-based drug being approved by the US Food and Drug Administration and the European Medicines Agency in 2018 [32]. No miRNA-based strategy has received regulatory approval yet; however, there are multiple candidates in preclinical or clinical stages for treating rare genetic diseases, cancer, and inflammatory disorders, amongst others [33]. Coupling RNAi with patient-specific disease models holds immense potential for the elucidation of complex disease pathology modifiers and in the area of oligonucleotides in therapeutic strategies. Herein, we report a simple user-friendly method that can be applied to different patient-derived lung spheroid models, making it a versatile approach for studying gene and miRNA function in 3D spheroids, thereby facilitating drug discovery and testing in relevant human in vitro systems. Some limitations of this study include differences in the transfection efficiency, whereby iBC spheroids had a significantly lower transfection efficiency compared to iAT2s, which might in part explain why we did not see a decrease in the *PTEN* mRNA levels with miR-21 overexpression. Therefore, further optimization of the transfection protocol for the iBC spheroid model is required. Additional transfection reagents and/or alternative delivery methods coupling the nucleic acid cargo with biocompatible nanoparticles as the delivery carriers [11,34,35] could also be investigated for this model.

In conclusion, we have demonstrated the feasibility of small RNA transfection of human iPSC-derived whole-lung spheroids without disturbing the 3D structure or environment, in addition to achieving functional effects of siRNA and miRNA transfection on their target mRNAs and proteins. Further development of this method will facilitate functional studies to complement ongoing research into iPSC-derived spheroid platforms and inform delivery of future therapeutic strategies utilizing small RNAs.

## Data Availability

The original contributions presented in this study are included in the article/Supplementary Materials. Further inquiries can be directed to the corresponding author. The raw data supporting the conclusions of this article will be made available by the authors on request.

## Supplementary Materials

The following supporting information can be downloaded at: https://www.mdpi.com/article/10.3390/biomedicines13102419/s1. Supplementary Figure S1. Gating strategy for measuring transfection efficiency via flow cytometry. (A) All events were analysed using SSC-A and FSC-H and gates were established to remove debris. Gates were established for single cells using FSC-H and FCS-A. Cells were incubated with calcein blue to stain live cells. Gates were established on live cell population, which was used for analysis of AF647 fluorescence (i.e., cells transfected with fluorescently labelled siRNA). Displayed here are representative images of (B) unlabelled NC siRNA, (C) AllStars Neg siRNA AF 647, and (D) the corresponding histograms for panel C.
