# Supplementary figures and images for: Successful Delivery of Small Non-Coding RNA Molecules into Human iPSC-Derived Lung Spheroids in 3D Culture Environment
Source: Biomedicines. 2025 Oct 3;13(10):2419. doi: 10.3390/biomedicines13102419 (PMC12561723; doi:10.3390/biomedicines13102419)

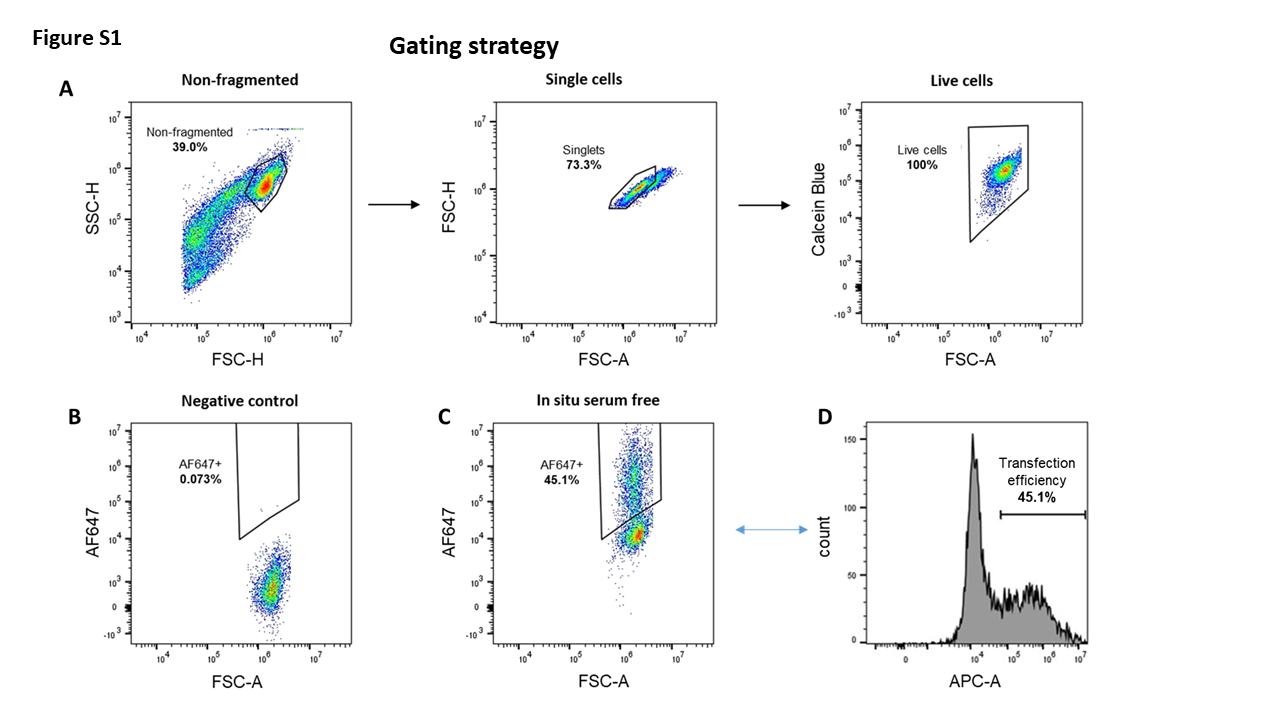

Supplement: Supplementary file 1 [file biomedicines-13-02419-s001.zip › biomedicines-3859699-supplementary.tif]
